# When is coercive methadone therapy justified?

**DOI:** 10.1111/bioe.12451

**Published:** 2018-06-08

**Authors:** Daniel D'Hotman, Jonathan Pugh, Thomas Douglas

**Keywords:** autonomy, criminal justice, drug policy, methadone, neuroethics

## Abstract

Heroin use poses a significant health and economic burden to society, and individuals with heroin dependence are responsible for a significant amount of crime. Owing to its efficacy and cost‐effectiveness, methadone maintenance therapy (MMT) is offered as an optional alternative to imprisonment for drug offenders in several jurisdictions. Some object to such ‘MMT offers’ on the basis that they involve coercion and thus invalidate the offender's consent to MMT. While we find these arguments unpersuasive, we do not attempt to build a case against them here. Instead, we explore whether administration of MMT following acceptance of an MMT offer might be permissible even on the assumption that MMT offers are coercive, and in such a way that the resulting MMT is non‐consensual. We argue that non‐consensual MMT following an MMT offer is typically permissible. We first offer empirical evidence to demonstrate the substantial benefits to the offender and society of implementing non‐consensual MMT in the criminal justice system. We then explore and respond to potential objections to such uses of MMT. These appeal respectively to harm, autonomy, bodily and mental interference, and penal theoretic considerations. Finally, we introduce and dismiss a potential response to our argument that takes a revisionist position, rejecting prevailing incarceration practices.

## 1 INTRODUCTION

The relationship between heroin dependence and crime is well documented.^1^ The United Nations estimates that the cost of drug‐related crime in the U.S.A. exceeds 1% of gross domestic product (GDP), equating to approximately US$160 billion each year,^2^ with heroin users accounting for a large proportion of this economic burden.^3^ Heroin users are also over‐represented in prison populations.^4^ Yet despite the efforts of law enforcement agencies, the health, social and economic costs associated with heroin dependence continue to increase.^5^

While scholars disagree about the goals of criminal justice, it is widely accepted that one of its objectives is to prevent convicted offenders from re‐offending — for both their own benefit and that of society.^6^ However, a significant body of evidence demonstrates that imprisoning drug offenders is ineffective at preventing recidivism or drug use.^7^ Methadone maintenance therapy (MMT), by contrast, has proven to be effective in reducing heroin use,^8^ and, in turn, drug‐related crime.^9^ As a result, it has been proposed as an optional alternative to incarceration for non‐violent drug offenders.^10^ For instance, in Australia, the treatment of drug offenders — including with MMT, counselling and psychotherapy — has been a popular alternative to incarceration for about 40 years, with judges frequently offering treatment to heroin‐dependent offenders in exchange for a reduced or commuted prison sentence if the prisoner accepts to undergo MMT.^11^ In light of the success of this policy, the U.K., China, and the majority of countries in the European Union have followed suit by implementing MMT in their own criminal justice systems.^12^

However, despite its success in preventing recidivism, attempts to establish MMT as a mainstay intervention for drug offenders have been held back by political opposition,^13^ preference for abstinence‐based treatments in the criminal justice system,^14^ and ethical protest from clinicians and philosophers.^15^

In this paper, we examine whether there is any sound ethical objection to the use of MMT in criminal justice systems on the model employed in Australia, among other countries. We argue that such use is morally permissible. After outlining a number of assumptions that we make throughout the paper (Section 2), we present the case for coercive MMT, offering empirical evidence for its potential to benefit both the offender and society (Section 3). We then go on to explore and respond to potential objections to our position in favour of coercive MMT (Section 4). These appeal respectively to harm, autonomy, bodily and mental interference, and penal theoretic considerations. Finally, we introduce, assess and ultimately reject a potential response to our argument according to which prevailing incarceration practices are unjustified (Section 5).

## 2 COERCION, COMPETENCE, AND THE CONSENT REQUIREMENT

We will use the term ‘the MMT offer’ to refer to the practice of offering MMT, with or without other forms of medical or psychological treatment, to criminal offenders in return for a shorter than normal prison term. One possible objection to the MMT offer claims that offering MMT in return for a reduction in prison term is coercive, thus rendering the recipient's consent to undergo MMT invalid. There is considerable philosophical literature examining whether an offer can be coercive in a manner that undermines the voluntariness of an individual's consent.^16^ However, the possibility of coercive offers has been raised in a wide range of contexts, including in debates on markets for human organs,^17^ the treatment of psychiatric diseases,^18^ research ethics,^19^ and the use of chemical castration in the criminal justice system.^20^

Many of the arguments made in these contexts seem applicable to the MMT offer. Consider, for example, William Green's claims regarding the practice of offering sexual offenders the opportunity to undergo so‐called ‘chemical castration’ in return for a reduced sentence. Green claims that, in the context of this offer,

Freedom of choice is impossible because the convict's loss of liberty constitutes a deprivation of such a magnitude that he cannot choose freely and voluntarily, but he is forced to give consent to an alternative he would not otherwise have chosen.^21^

Similarly, a heroin‐dependent offender facing a lengthy prison sentence might be understood as being ‘forced’ to give consent to MMT when he would not otherwise have done so. Accordingly, it seems plausible to suppose that some opponents of the MMT offer might claim that it is coercive.

We are sceptical about the plausibility of this claim; we agree with others that offers like the MMT offer are not coercive in a manner that would invalidate the consent of the recipient.^22^ Rather than attempting to make this case here, however, throughout the remainder of the article we assume that the MMT offer *is* coercive in a manner that invalidates the recipient's consent. Instead, we argue that it may be permissible to make the MMT offer *even if* it renders invalid the recipient's consent to undergo MMT. That is, we challenge the view that valid consent is required for the morally permissible imposition of MMT — a view we henceforth refer to as *the MMT consent requirement*.

The MMT consent requirement is consistent with views on consent taken elsewhere in medical ethics. MMT is a type of medical intervention, and it is widely held that it is permissible to carry out a medical intervention on a competent individual only if that individual provides valid consent to the intervention.^23^ We do not dispute the view that *most* medical interventions can permissibly be provided only with the valid consent of the patient. Rather, we argue that *the use of MMT within criminal justice* is, under certain conditions, a special case to which ordinary medical‐ethical consent requirements do not extend.

One way to reject the MMT consent requirement might be to deny that heroin‐dependent offenders are autonomous and thus maintain that they are not competent to provide (or refuse) informed consent to MMT.^24^ This is not our strategy; we assume that heroin‐dependent offenders can be competent to refuse consent to MMT.^25^ Rather, we tackle the MMT consent requirement head on, by arguing that we may have sufficient moral grounds to administer MMT to competent offenders even if their consent has been coerced and is invalid.

We also make one further significant assumption. David Wendler and Alan Wertheimer have recently argued that recruiting a person into medical research on the basis of coerced consent is morally worse than recruiting them into such research in the absence of consent.^26^ Were this position extrapolated from the context of medical research to the context of criminal justice, it might imply that it would be preferable to administer MMT to offenders without offering any alternative — that is, without soliciting their consent — than to adopt the coercive offer model. Whilst we believe that Wendler and Wertheimer plausibly defend their view insofar as it applies only to the specific context of research, we do not believe that it translates straightforwardly to the context of criminal justice. We cannot defend this belief here;^27^ instead, we simply assume, *contra* Wendler and Wertheimer, that acting on the basis of coerced consent is morally equivalent to acting in the absence of consent. Notably, if the reader finds this assumption problematic, rejecting this assumption would not render our arguments here obsolete. Rather, it would entail that they should simply be taken as arguments in favour of administering MMT without offering an alternative.

We shall now outline the case in favour of using MMT to reduce recidivism amongst drug offenders before considering objections to the non‐consensual administration of MMT.

## 3 THE CASE IN FAVOUR OF COERCIVE MMT

Methadone is an opioid used in the treatment of opiate addiction as part of MMT.^28^ Owing to its activation of endogenous opiate receptors (the pleasure receptors in the brain that are activated by heroin), methadone may be used as a substitution treatment for heroin addicts to prevent withdrawal, block the effects of heroin, and relieve narcotic cravings.^29^ Methadone is non‐sedating and non‐euphoric at stable doses, meaning that patients are able to work, drive, and feel a full range of emotions without narcotic impairment.^30^

Since the 1960s, a number of studies have been conducted to assess the effectiveness of MMT in treating heroin addiction. In a Cochrane review of 11 randomized control trials, MMT was more effective in retaining patients and supressing heroin use, and received better reviews in patient self‐reports, than drug‐free alternative treatments, including counselling and abstinence therapies.^31^ MMT reduces the spread of HIV and other infectious diseases, a trend attributed to a reduction in injecting, needle‐sharing, and associated high‐risk sexual activities (such as prostitution and unsafe sex).^32^ Enrolment in methadone programs has also been shown to cut mortality amongst enrolled heroin users by 50%^33^ and to substantially reduce criminal activity.^34^ For example, Lind et al. found that for every 100 persons enrolled in methadone programs for one year, New South Wales has 57 fewer break‐and‐enters, 56 fewer motor‐vehicle thefts, and 12 fewer robberies. Finally, MMT has been found to improve psychological wellbeing, employment opportunities and social functioning, and to reduce the societal costs of criminal behaviour and substance abuse.^35^

Admittedly, there are some weaknesses in the data; for example, there is limited evidence on MMT's long‐term effects.^36^ This gap in the literature may primarily be attributable to difficulties with long‐term patient retention and high drop‐out rates.^37^ Nevertheless, reviews conducted by the Institute of Medicine and National Institute of Health concluded that methadone is the most effective treatment for heroin addiction.^38^ Similarly, a common position paper commissioned by the Joint United Nations Programme on HIV/AIDS (UNAIDS), the World Health Organisation (WHO), and the United Nations Office on Drugs and Crime (UNODC) holds that

Substitution maintenance therapy is one of the most effective treatment options for opioid dependence. It can decrease the high cost of opioid dependence to individuals, their families and society at large by reducing heroin use, associated deaths, HIV risk behaviours and criminal activity. Substitution maintenance therapy is a critical component of community‐based approaches in the management of opioid dependence and the prevention of HIV infection among injecting drug users.^39^

Some have expressed concern that these positive effects of MMT have been identified through the study of *voluntary* MMT programs and would not translate to coercive contexts, as effective treatment requires motivation to change on behalf of the addicted person.^40^ However, multiple studies have concluded that patients who enter methadone programs under coercion perform as well as those who enter treatment voluntarily.^41^ In fact, some suggest that MMT may actually have greater effectiveness when there is some level of coercion involved, as patients are retained in the program for longer periods, an important factor for preventing relapse and recidivism.^42^ Despite these promising results, there remain few patients in coercive MMT programmes. Indeed, of the 1.5 million people arrested in the U.S.A. in 2008 who were at risk of drug abuse or dependence, only 3.8% actually received treatment.^43^

## 4 THE CASE AGAINST COERCIVE MMT

It might be argued that not all of the benefits of MMT have a bearing on the permissibility of using it coercively within the criminal justice system. For instance, some might advance the broadly Millian argument that coercive treatment can never be justified by its benefits to the treated individual, because individuals should always be regarded as the best judges of their own interests; coercing individuals into treatment for their own good amounts to wrongful paternalism.^44^ Alternatively, it might be claimed that, even if coercive treatment can sometimes be justified by reference to its benefits for the treated individual, benefitting the treated individual in the case of convicted drug offenders would be contrary to the putative retributive aims of criminal justice.

Note, however, that at least some of the benefits of MMT cited above are benefits to *others*. Moreover, some of these benefits to others — namely, those due to reduction in criminal recidivism — are benefits that would generally be regarded as among the justifying grounds for other forms of coercion within criminal justice systems. The prevention of recidivism, or the maintenance of public security that it serves, are generally taken to be at least part of what justifies coercive probation, incarceration and rehabilitation programs, and MMT is a relatively cost‐effective means of realizing this objective.^45^

Despite the strong case in favour of coercive MMT, arguments in favour of the practice must be weighed against the importance of respecting an offender's autonomy by administering medical treatments only with their valid consent. In their discussion of the principle of respect for autonomy, Beauchamp and Childress discuss the Kantian and Millian roots of the salience that we now attribute to individual autonomy.^46^ Interestingly though, Mill's defence of ‘individuality’ in *On Liberty* provides clues as to how it might be possible to overcome the presumption in favour of personal autonomy when we consider the permissibility of coercive MMT. In his famous ‘harm principle’, Mill claims:

The only purpose for which power can be rightfully exercised over any member of a civilized community, against his will, is to prevent harm to others.^47^

The harm principle is compatible with the view that the prevention of harm to others is insufficient to justify the imposition of involuntary medical interventions — it claims only that harm prevention is *necessary* for such a justification. However, it leaves open the possibility that the moral reason we have to prevent harm to others may be sufficient to justify the rightful exercise of power over the individual. This is important to acknowledge in the current context; the case of coercive MMT is not like common treatment scenarios in medical ethics in which considerations of personal autonomy are simply weighed against considerations of beneficence and non‐maleficence, and often understood to win out. In the current context, the principle of respect for autonomy must also be weighed against our moral reason to prevent harm to others (and, perhaps, other penal theoretic considerations, which we consider in further detail below).^48^

In fact, we already interfere with the autonomy of large numbers of heroin‐addicted criminal offenders by, for example, subjecting them to non‐consensual incarceration, probation arrangements, and community service. Moreover, it is widely (though not universally) accepted that at least a part of our reason for doing so is to prevent criminal recidivism, and thus harm to other members of society. Assuming that these other coercive measures can be justified for the purposes of preventing recidivism, the question is why coercive MMT could not be justified on similar grounds. In the following sections we shall approach this question from a number of perspectives in order to determine whether coercive MMT may be justified in a criminal justice setting.

First, however, a clarification. It might be held that, insofar as our argument for coercive MMT advocates sacrificing the prisoner's interests for the sake of the public good, it depends on the adoption of a utilitarian moral outlook according to which one individual's interests can be sacrificed whenever this would, in aggregate, confer a greater benefit on others. However, this is not so. Our argument is consistent with the existence of deontological constraints on the ways in which we may treat other people in order to promote the public good. Indeed, it is consistent even with the existence of deontological constraints that would ordinarily rule out the coercive imposition of medical treatments. It requires only that either (i) these constraints are non‐absolute, such that they can permissibly be infringed if the case for doing so is sufficiently strong, or (ii) individuals can make themselves liable to forms of treatment that would ordinarily violate those constraints (perhaps, for example, through committing a moral wrong).^49^

### 4.1 Harm

It would be difficult to reject coercive MMT by appealing to its harmful effects, while also accepting other coercive criminal justice practices. MMT has few negative side‐effects and is linked with positive outcomes after release; in typical cases it plausibly confers a net benefit on the treated individual. By contrast, many prevailing criminal justice practices have severe adverse side‐effects and plausibly cause net harm (indeed they may be *intended* to cause net harm, a point to which we will return below). Consider incarceration. This tends to be highly disruptive of social relationships and career projects, has a number of adverse health and social outcomes for drug offenders, and, as we have seen, is frequently ineffective in preventing recidivism. Moreover, incarceration may need to be continued for many years without abatement if *any* anti‐recidivist effect is to be maintained.^50^ In contrast, offenders may have to engage with mandated treatment for a much shorter period of time, owing to its efficacy as a rehabilitative measure. It would thus be difficult to eschew MMT while endorsing incarceration on the basis that the former is more harmful or offers a less favourable harm–benefit balance.

### 4.2 Autonomy

Similarly, those who accept other coercive measures for the purposes of recidivism‐prevention cannot object to coercive MMT solely on the basis that it is coercive or interferes with autonomy; incarceration, probation, and community service also involve serious affronts to autonomy and indeed would for this reason be regarded as completely unacceptable if they were imposed on innocent people. To the extent that these interventions are accepted, they are accepted in part because we think that criminal offenders have either made themselves liable to certain intrusions on their autonomy, or because the benefits of restricting offender autonomy are sufficiently weighty as to justify some infringements of the requirement to respect autonomy.

Of course, certain preferences may be more central to an agent's autonomy than others. For example, it might be argued that the aspects of autonomy interfered with by coercive MMT are objectively more important than the aspects of autonomy interfered with by other coercive interventions in criminal justice. We now turn to consider two arguments for this view.

### 4.3 Bodily interference

The first of these arguments appeals to the fact that coercive MMT involves bodily interference because it involves coercively introducing a pharmaceutical agent into the recipient's body. It is widely assumed that reasons to respect a person's autonomy entail reasons not to interfere with a person's body, and that these reasons apply irrespective of what consequences the interference may have, and what intentions motivate it.^51^ Importantly, this suggests that coercive MMT might be more morally problematic than incarceration even if it is less harmful to the offender, and overall less threatening to autonomy, because in interfering with the body, it interferes with one particularly important aspect of autonomy, namely, freedom from bodily interference.

However, it is not clear that freedom of bodily interference is *more* central to autonomy than the aspects of autonomy that are interfered with by other coercive criminal justice measures.^52^ For instance, freedom of movement and freedom of association are plausibly also particularly important aspects of autonomy, inter alia because of their importance for maintaining social relationships. This is why it is almost never permissible to severely constrain them and why practices that do severely constrain them (such as quarantine and psychiatric detention) are normally subject to extensive legal checks and balances.

Moreover, it is important here to note the difference between different degrees of bodily interference. It is plausible that constraints on bodily interference are more stringent in respect of more severe forms of interference (e.g., extreme violence) than in respect of less severe forms (e.g., minor, non‐sexual touching). Thus, whilst it seems that offenders should be protected against extreme violence, minor forms of bodily interference can plausibly be permissible. For instance, it seems plausible that offenders may permissibly be required to undergo a non‐consensual mouth swab to obtain DNA evidence. Coercively administering methadone arguably involves greater bodily interference than performing a non‐consensual mouth swab and there may thus be a more stringent constraint against such interference. Nevertheless, it is certainly not obvious that the constraint is more stringent than the constraints on, for example, the very extensive interference with freedom of movement and association entailed by incarceration.

### 4.4 Mental interference

At this point, opponents might protest that coercive MMT involves a further serious kind of intrusion that incarceration does not involve: it directly interferes with the mental life of the offender by biologically modulating the offender's mental states (for example, suppressing desires for heroin).

Mental interference has attracted scant attention in the philosophical literature, with little work having been done to establish its nature and moral status.^53^ Yet, it is plausible that non‐consensual mental interference can be wrong even in the absence of any harm, and even in contexts, such as criminal justice, where serious interference with autonomy is normally taken to be justified. Consider ‘brainwashing’, for example, through aversion therapy or hypnosis. Although not necessarily harmful, many would intuitively find such interventions to be morally problematic, even if used within a criminal justice system to prevent criminal recidivism. One plausible explanation for this would hold that these interventions involve an objectionable form of mental interference. Perhaps coerced MMT would be wrong for the same reason.^54^

However, we are commonly subjected to various forms of mental interference in our daily lives. Consider, for example, the use of randomized rewards to promote habitual engagement with computer games or social media platforms, the use of repeated temptation to undermine willpower in product marketing, and the use of salience effects to promote healthy dietary choices (for example, by serving food on smaller plates or placing healthier products at eye level). At least some of these forms of mental interference are widely thought to be morally permissible even though it is not — to us at least — clear that they involve a less serious form of mental interference than coercive MMT.

Of course, opponents of coercive MMT might simply choose to bite the bullet here and assert that both the use of such environmental interventions and coercive MMT are impermissible. However, this strategy becomes less appealing when we consider also the mental effects of the status quo method of dealing with drug offenders, namely, incarceration. By virtue of the fact that incarceration involves removing an offender's social and economic connections and placing him or her in an extremely hostile and dangerous environment (i.e., prison), both of which can cause substantial stress and distress, it seems plausible to claim that incarceration involves a more serious form of mental interference than coercive MMT.^55^ Assuming that incarceration is nevertheless permissible — an assumption that we will re‐visit in the next section — and assuming that the case in favour of coercive MMT is as strong as that in favour of incarceration, one would then need to identify some factor other than mental interference in virtue of which coercive MMT is not permissible.

This might seem too quick. There is some intuitive plausibility to the thought that there is a difference between eliciting mental changes via a pharmacological intervention and eliciting such changes via an effect on the individual's environment, even if the latter leads to greater harm. One way of cashing this thought out is to claim that MMT involves a *direct* modification of the subject's brain, whereas any neural effects of incarceration, product marketing and other environmental interventions are indirect, in the sense that they are mediated by psychological processes.^56^

However, even if there is a compelling descriptive difference between direct and indirect interventions of this sort, this distinction will only be of use to the opponent of MMT if the distinction also has *normative* significance.^57^ Though we cannot canvass all plausible routes to establishing its normative significance here, we remain sceptical of this claim. Focquaert and Schermer suggest that the direct/indirect distinction is morally relevant insofar as it tracks the distinction between (correspondingly) active interventions, which require specific psychological and/or behavioural efforts on behalf of the recipient to achieve the desired end of the intervention, and passive interventions, which bring about the desired end by themselves.^58^ In our view, incarceration is a counterexample to the claim that the directness of the intervention tracks the passivity of the intervention. Incarceration is clearly an indirect intervention in terms of evincing mental changes, but it is far from clear that these changes require specific psychological and/or behavioural efforts on behalf of the recipient. We suggest that the onus is on the opponent of MMT to provide an alternative explanation for why the direct/indirect distinction should be understood to matter morally. In the absence of such an explanation, the most morally significant consideration simply seems to be that the (indirect) adverse mental effects of incarceration far outweigh the (direct) adverse mental effects of MMT in terms of the harm caused.

We remain open to the possibility that future scientific and philosophical work will uncover a morally relevant difference between the kind of interference involved in intuitively permissible environmental interventions, on the one hand, and that involved in coercive MMT, on the other. This research may also uncover some reason why the seemingly minor mental interference associated with coercive MMT is morally worse than the seemingly significant mental interference involved in incarceration. However, pending such developments, it is at best unclear that an appeal to the wrongness of mental interference could establish a decisive case against coercive MMT.

### 4.5 Penal theoretic considerations

As mentioned above, the context we are considering is one in which the principle of respect for autonomy must be weighed against harms to society. However, it might be argued that penal theoretic considerations are also relevant and that these speak against coercive MMT. In this section, we will attempt to allay such concerns, arguing that coercive MMT is consistent with modern theories of punishment in criminal justice.

There is significant disagreement over what objectives the criminal justice system should adopt, let alone which of these should be prioritized. Nevertheless, most authors concur that criminal justice systems should realize one or more of:

- Retribution.^59^
- General deterrence (deterring offending by individuals besides the offender in question).^60^
- Preventing the criminal in question from reoffending — for example, through incapacitation, rehabilitation or ‘specific’ deterrence.^61^

Medical treatment‐based approaches are generally neglected in discussions of criminal justice reform, although they appear broadly consistent with the rehabilitation component of (C). One plausible reason for this is that they are regarded as at odds with (A) and (B). A critic of employing coercive MMT in criminal justice might worry that a solely treatment‐based intervention would be too ‘soft’ to effectively realize the retributive and deterrent goals of criminal justice.^62^

However, coercive MMT need not impede the realization of retributive and deterrent objectives.^63^ Any system that incorporated coercive MMT would likely also need to include at least a short period of detainment in order to ensure compliance to the treatment model. Moreover, coercive MMT could be supplemented with further independent measures, such as community service or fines, intended to achieve retributive and deterrent goals. Thus, even if coercive MMT were to be classified as a solely rehabilitative measure, courts could mandate community service or issue fines to satisfy deterrent and retributive goals. In light of this, it is not clear that a requirement for deterrence or retribution alone offers a compelling argument against coercive MMT.

Still, to the extent that the objectives of criminal justice are limited to deterrence and retribution, it might be objected that there is no positive case for deploying MMT within criminal justice.^64^ However, we note that few seem prepared to endorse a conception of criminal justice that entirely excludes objectives other than deterrence and retribution.^65^ Parole boards frequently place stringent conditions on offenders that must be met in order to avoid a return to prison, and these are generally set in order to reduce the risk of recidivism through rehabilitation, incapacitation, or a mixture of the two. For instance, sex offenders on parole are often restricted from residing within certain distances of schools, parks or playgrounds where children are likely to be.^66^ In addition, the implementation of anger management courses, as part of parole conditions for violent offenders, offers a prime example of a criminal justice intervention where rehabilitation plays a central role.^67^ These practices are widely accepted as legitimate components of criminal justice.

A less extreme retributivist might raise concerns over whether coercive MMT could respect the proportionality requirement(s) incorporated into retributivist theories. To see why, it is prudent to begin by distinguishing between positive and negative retributivism in the context of proportionality. Negative retributivism places only an upper limit on the severity of the punishment — it holds that punishment may be less‐than‐proportionate, but not more‐than‐proportionate, to the offender's culpability in respect of the crime. Positive retributivism places both a lower and an upper limit, holding that punishment may be neither less nor more than would be proportionate.^68^ With this distinction in mind, suppose that sentences currently given to offenders are as short as they can be without violating the lower proportionality limit; shortening these sentences and providing MMT instead, which, we assume, would reduce harm to the offender, would render the overall criminal justice response less‐than‐proportionate, even if it retains a period of incarceration. Note that this would not be a problem on negative retributivism. Yet even positive retributivists could plausibly accept other less costly and safer penalties that we discussed previously, such as fines or community service, to achieve proportionality.^69^ In light of this, it seems implausible that coercive MMT would necessarily result in a violation of the proportionality requirement incorporated into positive retributivist theories of punishment.

## 5 THE REVISIONIST RESPONSE

Some of our arguments above have taken as a start point the permissibility of prevailing criminal justice practices, including incarceration. For instance, we suggested above that *if* incarceration is permissible, it would be difficult to reject coerced MMT as impermissible on the grounds that it causes harm to the offender or infringes on autonomy.

One possible response to these arguments would be to hold that incarceration is in fact unjustified.^70^ In support, it might be noted that the harsh conditions prevalent in modern prisons often pose a serious risk to offenders’ physical and mental wellbeing. Richard Lippke (2007) argues that punishment of serious criminals should only require the ‘minimum conditions of confinement’, and that incarceration in its current form may not be justified for many offenders.^71^ Victor Tadros (2001) develops this view further, suggesting that any benefits of incarceration are often eclipsed by the relative harm it causes.^72^ If incarceration is indeed morally impermissible, our above responses to objections from harm and restrictions on autonomy would be undermined.

Note, however, that even Lippke and Tadros, both staunch critics of modern incarceration, limit their critiques to *prevailing* incarceration practices. Both have accepted that a less intrusive and less harmful form of incarceration could be justified.^73^ Yet even the most minimal intervention deserving of the name ‘incarceration’ would surely involve significant harms, for example through disrupting familial, romantic and other social relationships, and significant infringements of autonomy, for example through limiting freedom of movement and association.^74^ Coerced MMT will typically be less harmful than even minimal incarceration, and it is not clear that it involves a greater infringement of autonomy or a greater degree of mental interference. This suggests that those of our arguments that rely on a comparison between incarceration and coerced MMT will succeed even if only the most minimal forms of incarceration are permissible.

Another version of this revisionist response would hold that incarceration may be justified for certain crimes, but that non‐violent drug offences are not among them. This response could be grounded on the reasonable suggestion that coercive means of rehabilitation can be justified only if non‐coercive means are less effective. Putting possible broader economic and social benefits aside, there is evidence that the legalisation of drugs may facilitate an environment where treatment is more accessible to drug users and there is less incentive to commit crime. In the Netherlands, for example, long‐term heroin users are provided with free heroin by the government, provided that they attend medical clinics and make use of government housing. This program has reduced criminality, improved health outcomes, and reduced the economic costs associated with drug abuse.^75^ If public policy‐makers pursued such programs and legalized drugs, coercive practices such as incarceration and coercive MMT might become unnecessary for the achievement of optimal rehabilitation, and thus unjustifiable. There may well come a time when governments around the world choose to treat drug users in the health system and without coercion in ways that render coercive criminal justice responses — or at least the rehabilitative aspects of them — redundant. Yet, we doubt the political feasibility of any such approach in the short and medium term. So long as coercive criminal justice responses remain necessary to optimal rehabilitation, the arguments that we have presented in favour of coercive MMT will hold.

## 6 CONCLUSION

Methadone programs reduce the severe health risks associated with heroin dependence, assist drug offenders in achieving broad psychosocial change, and achieve lasting criminal reform. In the past, implementation of these programs has been marred by controversy and protest by those who hold coercive treatment to be impermissible. We have outlined an ethical case in favour of such programs and responded to several possible objections. Contingent on encouraging results from further enquiry into the issue of mental interference, we hope that the findings outlined in this paper will help to stimulate the development of more defensible public policy in this area.

## CONFLICT OF INTEREST

The authors declare no conflict of interest.

## Footnotes

1
McGlothlin, W. H., Anglin, M. D., & Wilson, B. D. (1978). Narcotic addiction and crime. *Criminology, 16*(3), 293–316.

2
United Nations Office on Drugs and Crime (UNODC). (2012). *World drug report*: *2012* Retrieved from https://www.unodc.org/documents/data-and-analysis/WDR2012/WDR_2012_web_small.pdf [Accessed 22 Nov, 2017]; Emery Jr., R. E., & Oltmanns, T. F. (1999). *Essentials of abnormal psychology*. London: Pearson.

3
Mark, T. L. , Woody, G. E., Juday, T., & Kleber, H. D. (2001). The economic costs of heroin addiction in the United States. *Drug and Alcohol Dependence, 61*(2), 195–206; United Nations Office on Drugs and Crime, *op. cit*. note 2. 10.1016/s0376-8716(00)00162-911137285

4
Hall, W. (1997). The role of legal coercion in the treatment of offenders with alcohol and heroin problems. *Australian & New Zealand Journal of Criminology, 30*(2), 103–120.

5
United Nations Office on Drugs and Crime (UNODC). (2014). *World drug report: 2014* Retrieved from https://www.unodc.org/documents/wdr2014/World_Drug_Report_2014_web.pdf [Accessed 22 Nov, 2017].

6
For the classical view, see De Beaumont, G. A., & De Tocqueville, A. (1968) [1833]. *On the penitentiary system in the United States: And its application in France; with an Appendix on penal colonies, and also, statistical notes*. New York, NY: Augustus M. Kelly.

7
Farrell, M., Ward, J., Mattick, R., Hall, W., Stimson, G. V., des Jarlais, D., … Strang, J. (1994). Fortnightly review: Methadone maintenance treatment in opiate dependence: A review. *BMJ, 309*(6960), 997–1001. 10.1136/bmj.309.6960.997PMC25413127950725

8
Ibid.

9
Lind, B., Chen, S., Weatherburn, D., & Mattick, R. (2005). The effectiveness of methadone maintenance treatment in controlling crime: An Australian aggregate‐level analysis. *The British Journal of Criminology, 45*(2), 201–211.

10
United Nations Office on Drugs and Crime (UNODC). (2013). *World drug report: 2013* Retrieved from https://www.unodc.org/unodc/secured/wdr/wdr2013/World_Drug_Report_2013.pdf [Accessed Nov 22, 2017]; Hall, *op. cit*. note 4.

11
Hall, W., & Lucke, J. (2010). Legally coerced treatment for drug using offenders: Ethical and policy issues. *Crime and Justice Bulletin, 144*, 1–12.

12
Schaub, M. , Stevens, A., Berto, D., Hunt, N., Kerschl, V., McSweeney, T., … Uchtenhagen, A. (2010). Comparing outcomes of ‘voluntary’ and ‘quasi‐compulsory’ treatment of substance dependence in Europe. *European Addiction Research, 16*(1), 53–60. 10.1159/00026593820016186

13
Fischer, B. (2000). Prescriptions, power and politics: The turbulent history of methadone maintenance in Canada. *Journal of Public Health Policy, 21*(2), 187–210. 10881454

14
Leukefeld, C. G., & Tims, F. (1988). Compulsory treatment: A review of findings. In C. G. Leukefeld & F. Tims (Eds.), *Compulsory treatment of drug abuse: Research and clinical practice (NIDA Research Monograph 86)* (pp. 236–254). Rockville, MD: U.S. Department of Health and Human Services.

15
Farabee, D., Prendergast, M., & Anglin, M. D. (1998). The effectiveness of coerced treatment for drug‐abusing offenders. *Federal Probation, 62*(1), 3–10.

16
See for example, Feinberg, J. (1989). *The moral limits of the criminal law. Volume 3: Harm to self* Oxford: Oxford University Press; Wertheimer, A. (1987). *Coercion* Princeton, NJ: Princeton University Press; Nozick, R. (1969). Coercion. In S. Morgenbesser, P. Suppes & M. G. White (Eds.), *Philosophy, science, and method: Essays in Honor of Ernest Nagel* (pp. 440–472). New York, NY: St Martin's Press.

17
Rippon, S. (2012). Imposing options on people in poverty: the harm of a live donor organ market. *BMJ, 40*(3), 145–150. 10.1136/medethics-2011-10031822745109

18
Shaw, J. (2007). Is it acceptable for people to be paid to adhere to medication? No. *BMJ, 335*(7613), 233. 10.1136/bmj.39286.422639.BEPMC193975417673765

19
Macklin, R. (1989). The paradoxical case of payment as benefit to research subjects. *IRB: Ethics & Human Research, 11*(6), 1–3. 11650284

20
Vanderzyl, K. A. (1994). Castration as an alternative to incarceration: An impotent approach to the punishment of sex offenders. *Northern Illinois University Law Review, 15*(1), 107–140.

21
Green, W. (1986). Depo‐Provera, castration, and the probation of rape offenders. *University of Dayton Law Review, 12*(1), 16–17.

22
Wertheimer, A., & Miller, F. G. (2014). There are (STILL) no coercive offers. *Journal of Medical Ethics, 40*(9), 592–593; Bomann‐Larsen, L. (2013). Voluntary rehabilitation? On neurotechnological behavioural treatment, valid consent, and inappropriate offers. *Neuroethics, 6*(1), 65–77. 10.1007/s12152-011-9105-9PMC360261723525251

23
Beauchamp, T. L., & Childress, J. F. (2012). *Principles of biomedical ethics* (7th ed.). Oxford: Oxford University Press.

24
National Bioethics Advisory Commission. (1998). *Research involving persons with mental disorders that may affect decisionmaking capacity* Retrieved from https://bioethicsarchive.georgetown.edu/nbac/capacity/TOC.htm [Accessed Nov 22, 2017].

25
Foddy, B., & Savulescu, J. (2006). Addiction and autonomy. *Bioethics, 20*(1), 1–15. 10.1111/j.1467-8519.2006.00470.x16680876

26
Wendler, D. & Wertheimer, A. (2017) Why is coerced consent worse than no consent and deceived consent?’ *The Journal of Medicine & Philosophy*, 42(2), 114–131. 10.1093/jmp/jhw064PMC590108828201684

27
Briefly, one significant problem is that Wendler and Wertheimer argue that this view requires researcher‐centric justification, which appeals to norms that pertain to how researchers ought to treat research subjects, but that are independent of what would best protect or respect those subjects. The problem with extending this argument beyond Wendler and Wertheimer's specified context of medical research is that many of the norms they appeal to in order to ground this view (such as the practicability condition) are quite specific to that context, and do not have an obvious corollary in non‐research contexts.

28
Chou, R., Cruciani, R. A., Fiellin, D. A., Compton, P., Farrar, J. T., Haigney, M. C., … Zeltzer, L. (2014). Methadone safety: A clinical practice guideline from the American Pain Society and College on problems of drug dependence, in collaboration with the Heart Rhythm Society. *The Journal of Pain, 15*(4), 321–337. 10.1016/j.jpain.2014.01.49424685458

29
Farrell et al., *op. cit*. note 7.

30
Joseph, H., Stancliff, S., & Langrod, J. (2000). Methadone maintenance treatment (MMT): A review of historical and clinical issues. *Mount Sinai Journal of Medicine, 67*(5–6), 347–364; Institute of Medicine. (1995). *Federal regulation of methadone treatment*. Washington, DC: The National Academies Press. 11064485

31
Mattick R. P., Breen, C., Kimber, J., & Davoli, M. (2009). Methadone maintenance therapy versus no opioid replacement therapy for opioid dependence. *The Cochrane Database of Systematic Reviews, 3*, CD002209. 10.1002/14651858.CD00220912519570

32
Gowing, L., Farrell, M. F., Bornemann, R., Sullivan, L. E., & Ali, R. (2011). Oral substitution treatment of injecting opioid users for prevention of HIV infection. *The Cochrane Database of Systematic Reviews, 8*, CD004145. 10.1002/14651858.CD004145.pub318425898

33
Gibson, A., Degenhardt, L., Mattick, R. P., Ali, R., White, J., & O’Brien, S. (2008). Exposure to opioid maintenance treatment reduces long‐term mortality. *Addiction, 103*(3), 462–468; Clausen, T., Anchersen, K., & Waal, H. (2008). Mortality prior to, during and after opioid maintenance treatment (OMT): A national prospective cross‐registry study. *Drug and Alcohol Dependence, 94*(1–3), 151–157. 10.1016/j.drugalcdep.2007.11.00318155364

34
Lind, B., et al. *op. cit.* note 9

35
Gibson, D. R., Flynn, N. M., & McCarthy, J. J. (1999). Effectiveness of methadone treatment in reducing HIV risk behavior and HIV seroconversion among injecting drug users. *AIDS, 13*(14), 1807–1818. 10.1097/00002030-199910010-0000210513638

36
Ball, J. C., & Ross, A. (1991). *The effectiveness of methadone maintenance treatment: patients, programs, services, and outcome*. London: Springer‐Verlag.

37
Zhou, K., & Zhuang, G. (2014). Retention in methadone maintenance treatment in mainland China, 2004–2012: A literature review. *Addictive Behaviors, 39*(1), 22–29. 10.1016/j.addbeh.2013.09.00124090627

38
Joseph, et al., *op. cit* note 30; Institute of Medicine, *op. cit*. note 30.

39
Lawrinson, P., Ali, R., Buavirat, A., Chiamwongpaet, S., Dvoryak, S., Habrat, B., … Zhao, C. (2008). Key findings from the WHO collaborative study on substitution therapy for opioid dependence and HIV/AIDS. *Addiction, 103*(9), 1484–1492. 10.1111/j.1360-0443.2008.02249.x18636999

40
Hartjen, C. A., Mitchell, S. M., & Washburne, N. F. (1981). Sentencing to therapy: Some legal, ethical, and practical issues. *Journal of Offender Counselling, Services & Rehabilitation, 6*(1–2), 21–39.

41
Brecht, M. L., Anglin, M. D., & Wang, J. C. (1993). Treatment effectiveness for legally coerced versus voluntary methadone maintenance clients. *The American Journal of Drug and Alcohol Abuse, 19*(1), 89–106. 10.3109/009529993090026688438834

42
Farabee, *op. cit*. note 15.

43
Rich, J. D., McKenzie, M., Shield, D. C., Wolf, F. A., Key, R. G., Poshkus, M., & Clarke, J. (2005). Linkage with methadone treatment upon release from incarceration: A promising opportunity. *Journal of Addictive Diseases, 24*(3), 49–59. 10.1300/J069v24n03_0416186082

44
Mill, J. S. (2015) [1859]. On liberty. In M. Philp & F. Rosen (Eds.), On liberty, utilitarianism, and other essays. Oxford: Oxford University Press; Dworkin, G. (1972). Paternalism. *The Monist, 56*(1), 64–84.

45
Farrell, M., Gowing, L., Marsden, J., Ling, W., & Ali, R. (2005). Effectiveness of drug dependence treatment in HIV prevention. *International Journal of Drug Policy, 16*(1), 67–75; Gerstein, D. R., & Harwood H. J. (Eds.). (1990). *Treating drug problems. Volume 1: A study of effectiveness and financing of public and private drug treatment systems*. Washington, DC: National Academy Press.

46
Beauchamp & Childress, *op. cit*. note 23.

47
Mill, *op. cit*. note 44, p. 30.

48
Ryberg, J. (2015). Is coercive treatment of offenders morally acceptable? On the deficiency of the debate. *Criminal Law & Philosophy, 9*(4), 619–631.

49
Pugh, J., & Douglas, T. (2016). Justifications for non‐consensual medical intervention: from infectious disease control to criminal rehabilitation. *Criminal Law & Philosophy, 35*(3), 205–229. 10.1080/0731129X.2016.1247519PMC531279628260832

50
Douglas, T. (2014). Criminal rehabilitation through medical intervention: Moral liability and the right to bodily integrity. *The Journal of Ethics, 18*(2), 101–122. 10.1007/s10892-014-9161-6PMC408326625009441

51
Ibid.

52
Ibid.

53
For an initial outline of the right to mental integrity, see: Douglas, *op. cit* note 50; Bublitz, J. C., & Merkel, R. (2014). Crimes against minds: On mental manipulations, harms and a human right to mental self‐determination. *Criminal Law & Philosophy, 8*(1), 51–77.

54
Douglas, *op. cit*. note 50.

55
This claim might be disputed on the basis that (many of) the mental effects of incarceration are unintended; it might be held that mental interference necessarily involves intentional modulation of mental states or processes, or that it is other‐things‐being‐equal more serious when its mental effects are intended.

56
Focquaert, F., & Schermer, M. (2015). Moral enhancement: Do means matter morally?, *Neuroethics, 8*(2), 139–151, p. 144.

57
This is essentially the challenge laid down by Neil Levy's ‘parity principle’. See Levy, N. (2007). *Neuroethics*. Cambridge: Cambridge University Press.

58
Focquaert & Schermer, *op. cit*. note 56.

59
Brooks, T. (2012). *Punishment* Abingdon, Oxon: Routledge; Boonin, D. (2008). *The problem of punishment*. Cambridge: Cambridge University Press.

60
Brooks, *op. cit*. note 59.

61
Tadros, V. (2013). *The ends of harm: The moral foundations of criminal law*. Oxford: Oxford University Press.

62
Ibid.

63
Brooks, *op. cit* note 59; Lippke, R. (2007). *Rethinking imprisonment*. Oxford: Oxford University Press.

64
Ryberg, J. (2012). Punishment, pharmacological treatment, and early release. *International Journal of Applied Philosophy, 26*(2), 231–244.

65
Levy, K. (2014). Why retributivism needs consequentialism: the rightful place of revenge in the criminal justice system. *Rutgers Law Review*, *66*(3), 629–684.

66
Levenson, J. S., & Cotter, L. P. (2005). The impact of sex offender residence restrictions: 1,000 feet from danger or one step from absurd? *International Journal of Offender Therapy and Comparative Criminology, 49*(2), 168–178; Burchfield, K. B., & Mingus, W. (2008). Not in my neighborhood assessing registered sex offenders’ experiences with local social capital and social control. *Criminal Justice and Behavior, 35*(3), 356–374. 10.1177/0306624X0427130415746268

67
Mcsherry, B., & Freckelton, I. (Eds). (2013). *Coercive care: Rights, law and policy*. Abingdon, Oxon: Routledge. For another example of this practice, see: L vs. RIDCA Central (Regional Disability Care Agency: 2010).

68
Von Hirsch, A., & Ashworth, A. (2005). *Proportionate sentencing: Exploring the principles*. Oxford: Oxford University Press.

69
Jesper Ryberg takes the argument one step further, claiming that a medical intervention alone could fulfil the proportionality requirement by virtue of the nature of the inconvenience and unpleasant sensations that it inflicts on an offender. See: Ryberg, *op. cit*. note 64.

70
See, for example, Barn, G. (2016). Can medical interventions serve as 'criminal rehabilitation'? *Neuroethics*. Doi: 10.1007/s12152‐016‐9264‐9. 10.1007/s12152-016-9264-9PMC641167530956727

71
Lippke, *op. cit*. note 63.

72
Tadros, *op. cit*. note 61.

73
Ibid.; Lippke, *op. cit*. note 63.

74
Douglas, *op. cit*. note 50.

75
Fischer, B., Oviedo‐Joekes, E., Blanken, P., Haasen, C., Rehm, J., Schechter, M. T., … van den Brink, W. (2007) Heroin‐assisted treatment (HAT) a decade later: A brief update on science and politics. *Journal of Urban Health, 84*(4), 552–562. 10.1007/s11524-007-9198-yPMC221955917562183

